# Hierarchical Characteristics of Dementia Community Support in Community General Support Centers

**DOI:** 10.1093/geroni/igaf122.2921

**Published:** 2025-12-31

**Authors:** Taeko Nakashima, Kei Sugiyama, Etsuyo Kamiyamasaki

**Affiliations:** Nihon Fukushi University, East Greenbush, New York, United States; Osaka Metropolitan University, Osaka, Osaka, Japan; Nihon Fukushi University, MIhama, Aichi, Japan

## Abstract

**Objective:**

This study aims to evaluate the community work practices of professionals engaged in dementia care at Community General Support Centers using the Latent Rank Theory and to clarify the hierarchical characteristics of their implementation.

**Background:**

In Japan, the Basic Act on Dementia to Promote an Inclusive Society, was enacted in 2024. This law aims to foster safe communities where people with dementia can live independently and securely with others.

**Methods:**

A questionnaire survey was conducted at 2,908 Community General Support Centers across 32 prefectures, with responses obtained from 876 professionals. After excluding cases with missing data, 515 responses were analyzed. The analysis utilized items related to dementia community work practices and was conducted based on the Latent Rank Theory.

**Results:**

Preliminary results suggest that a four-rank model best represents the practice levels. Support initiatives targeting commercial and public facility employees were generally low across all ranks. In Rank 1, items related to engaging with these sectors scored below 2 (“rarely implemented”), except for identifying potential collaborators. On the other hand, initiatives involving multidisciplinary professionals and local residents were recognized to some extent, even in the lower ranks, with a particularly strong presence in higher-ranked groups.

**Discussion:**

The importance of engaging with commercial and public facility employees is recognized among dementia community support professionals; however, actual efforts remain insufficient. To enhance these practices, it is essential to establish clear strategies for collaborating with these stakeholders.

